# Live imaging reveals chromatin compaction transitions and dynamic transcriptional bursting during stem cell differentiation in vivo

**DOI:** 10.7554/eLife.83444

**Published:** 2023-03-07

**Authors:** Dennis May, Sangwon Yun, David G Gonzalez, Sangbum Park, Yanbo Chen, Elizabeth Lathrop, Biao Cai, Tianchi Xin, Hongyu Zhao, Siyuan Wang, Lauren E Gonzalez, Katie Cockburn, Valentina Greco

**Affiliations:** 1 Department of Genetics, Yale University School of Medicine New Haven United States; 2 Institute for Quantitative Health Science & Engineering (IQ), Michigan State University East Lansing United States; 3 Division of Dermatology, Department of Medicine, College of Human Medicine, Michigan State University East Lansing United States; 4 Department of Pharmacology and Toxicology, College of Human Medicine, Michigan State University East Lansing United States; 5 Department of Biostatistics, Yale University School of Public Health New Haven United States; 6 Deparment of Cell Biology, Yale University School of Medicine New Haven United States; 7 Department of Biochemistry and Rosalind & Morris Goodman Cancer Institute, McGill University Montreal Canada; 8 Departments of Cell Biology and Dermatology, Yale Stem Cell Center, Yale Cancer Center, Yale University School of Medicine New Haven United States; University of Pennsylvania United States; Max Planck Institute for Heart and Lung Research Germany

**Keywords:** stem cells, chromatin, live imaging, transcription, differentiation, Mouse

## Abstract

Stem cell differentiation requires dramatic changes in gene expression and global remodeling of chromatin architecture. How and when chromatin remodels relative to the transcriptional, behavioral, and morphological changes during differentiation remain unclear, particularly in an intact tissue context. Here, we develop a quantitative pipeline which leverages fluorescently-tagged histones and longitudinal imaging to track large-scale chromatin compaction changes within individual cells in a live mouse. Applying this pipeline to epidermal stem cells, we reveal that cell-to-cell chromatin compaction heterogeneity within the stem cell compartment emerges independent of cell cycle status, and instead is reflective of differentiation status. Chromatin compaction state gradually transitions over days as differentiating cells exit the stem cell compartment. Moreover, establishing live imaging of *Keratin-10* (*K10*) nascent RNA, which marks the onset of stem cell differentiation, we find that *Keratin-10* transcription is highly dynamic and largely precedes the global chromatin compaction changes associated with differentiation. Together, these analyses reveal that stem cell differentiation involves dynamic transcriptional states and gradual chromatin rearrangement.

## Introduction

Cellular identity is a composite of many features, including behavior, morphology, protein levels, and gene expression. All of these aspects are fundamentally shaped by the transcriptional program and therefore chromatin architecture of a cell. Recent technological advances have allowed the field to increasingly appreciate transcriptional heterogeneity within cell populations that were previously assumed to be homogeneous (Patel et al., 2014; Wang et al., 2020), and single-cell epigenetic profiling is beginning to reveal the extent of chromatin architecture heterogeneity within cell populations (Buenrostro et al., 2015; Finn et al., 2019; Jin et al., 2015; Lai et al., 2018).

The particular suite of genes expressed in a given cell is largely determined by nucleosome compaction in different regions of the genome, where accessible (euchromatic) regions permit gene expression, and inaccessible (heterochromatic) regions largely prevent gene expression. During embryonic lineage specification, stem cell differentiation, and somatic cell reprogramming, chromatin architecture undergoes large-scale changes resulting in drastically different cell identities (Golkaram et al., 2017; Kurimoto et al., 2015; Oudelaar et al., 2020; Paulsen et al., 2019; Pelham-Webb et al., 2020). However, because chromatin architecture analyses, including those with single-cell resolution, typically rely on data captured at fixed timepoints, we lack an understanding of how chromatin architecture progressively changes during cell identity transitions within a physiological setting.

Epidermal stem cell differentiation is an excellent model to understand the progressive nature of cell identity transitions. The epidermis is fueled by a basal layer of stem cells which are actively proliferating and continually differentiating apically to build the outer layers of the skin barrier. (Figure 1—figure supplement 1A). Traditionally, cell identities within the epidermis have been distinguished by cell morphology, specific protein markers, and localization within the tissue.

Recently, single-cell RNA-sequencing data has shown that the cell identity transition through epidermal differentiation is progressive and takes place over several days. Specifically, cells within the basal stem cell layer show global transcriptional changes associated with differentiation preceding exit from the basal layer (Aragona et al., 2020; Cockburn et al., 2022). Chromatin accessibility and the architecture of individual loci have been investigated in embryonic skin (Fan et al., 2018; Gdula et al., 2013; Mardaryev et al., 2014; Shue et al., 2020), but how and when chromatin changes relative to the transcriptional and morphological transitions of adult epidermal stem cell differentiation remains unknown.

Here, we leverage intravital imaging to observe and track global chromatin changes of individual stem cells within their homeostatic environment through time and cell fate transitions. By developing a quantitative pipeline to capture each cell’s unique chromatin compaction state, we reveal extensive heterogeneity of global chromatin architecture within the epidermal stem cell population, as well as distinct chromatin compaction states of epidermal stem cells and their differentiated daughter cells. Tracking individual cells over time and using a reporter for differentiation status reveals that global chromatin compaction state reflects differentiation state, beginning in the basal layer prior to exit from the stem cell compartment. We also show, through live imaging endogenous transcription at the earliest known stage of differentiation, that epidermal cells pass through heterogeneous and flexible transcriptional states as they progress towards their fully differentiated status. Together, this study reveals the chromatin compaction heterogeneity within a regenerative organ, incremental chromatin compaction remodeling through stem cell differentiation, and insight into how transcriptional dynamics of a key differentiation gene relate to cellular state transitions.

## Results

### Intravital imaging reveals cell-cycle-independent heterogeneity in chromatin compaction across the basal stem cell layer

**Figure 1.**
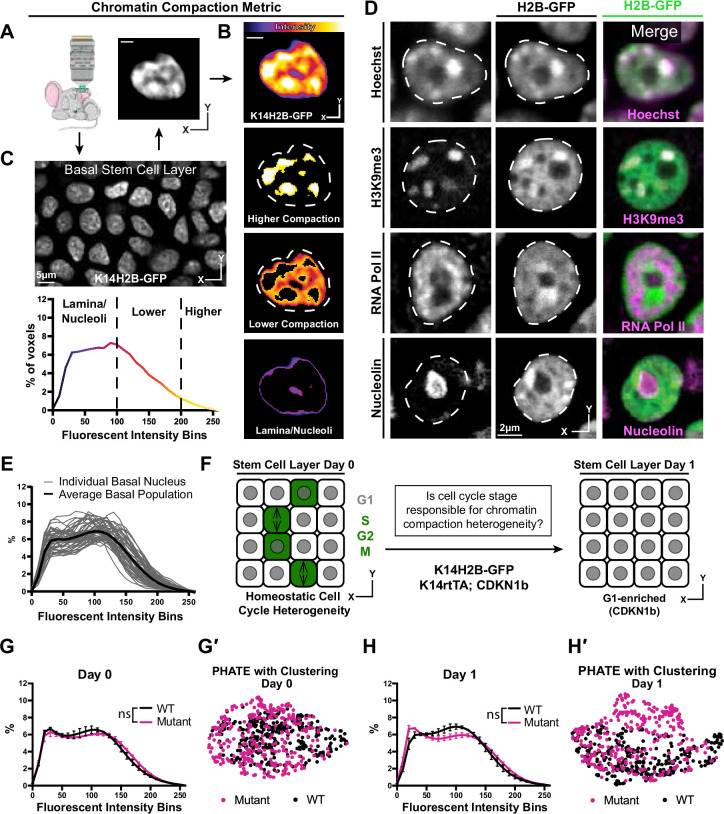
Chromatin compaction state is heterogeneous and independent of interphase cell cycle. (**A**) Representative XY view of the basal stem cell layer showing the *Kertain14*-driven Histone2B-GFP allele in a live mouse. (**B**) A representative chromatin compaction profile of a single basal stem cell nucleus. Each voxel from the 3D volume of a nucleus was exported, normalized for mean fluorescent intensity, and plotted as a voxel percentage of volume against the 0–256 intensity bins (methods). Single optical slice. (**C**) A representative nucleus expressing H2B-GFP in a single optical slice, where the fluorescence intensity is displayed as a heatmap to illustrate the range of chromatin compaction within a nucleus. See Video 1 for a 3D rendering of this nucleus. (**D**) Fixed epidermal tissue expressing H2B-GFP (green) and co-stained with Hoechst or various subnuclear compartment markers (magenta), demonstrating that high H2B-GFP fluorescence intensity correlates with heterochromatin (H3K9me3), lower H2B-GFP fluorescence intensity correlates with euchromatin (RNA Pol II), and the lowest H2B-GFP fluorescence intensity (in part) correlates with nucleoli (Nucleolin). The colocalization/overlap of these two fluorophores are indicated by the presence of white signal. Nuclear outlines are traced in white, dotted lines. Single optical slice. (**E**) Chromatin compaction plots for 50 individual basal stem cells (grey lines) and the averaged population (bold, black line) revealing substantial heterogeneity of chromatin compaction states within the stem cell population. (**F**) Schematic of the genetic p27 (*Cdkn1b*) overexpression system to stall cells in late G1 after 1 day of doxycycline administration. An increased N of 1260 basal nuclei across all mice (3 mutant and 3 wild-type) over day 0 and day 1 because only a subset of basal stem cells are within a non-G1 cell cycle phase at any given time. (**G**) Comparison of chromatin compaction states of wild-type (K14H2B-GFP; K14rtTA) and mutant (K14H2B-GFP; K14rtTA; tetO-Cdkn1b) mice prior to doxycycline administration/induction on day 0. (**G′**) PHATE plot of the same wild-type and mutant cells from panel (**G**) on day 0 showing intermixed populations. (**H**) Comparison of chromatin compaction states of wild-type (K14H2B-GFP; K14rtTA) and mutant (K14H2B-GFP; K14rtTA; tetO-Cdkn1b) mice one day post doxycycline induction and stalling of the cell cycle in late G1 showing non-significant changes between wild-type and mutant populations. (**H′**) PHATE plot of the same wild-type and mutant cells from panel (**H**) on day 1 after Cdkn1b induction showing largely intermixed populations. Mean and standard deviation among mice shown in panels G and H. Figure 1—source data 1.Heterogeneous chromatin compaction independent of interphase cell cycle status.

To understand transitions in large-scale chromatin architecture as a function of cell identity, we developed a fluorescence-based system that allows visualization of chromatin compaction in single skin epidermal stem cells within their native tissue in live mice. This quantitative pipeline leverages fluorescently-tagged histone 2b where bright fluorescence indicates densely packed chromatin and dimmer fluorescence indicates loosely packed chromatin or chromatin-excluded compartments (Amiad-Pavlov et al., 2021; Kanda et al., 1998). Using the Keratin-14 histone2B-GFP (K14H2B-GFP) allele, which is expressed in epidermal stem cells and their progeny, we first segmented the 3D volume of individual nuclei and extracted fluorescence intensity at each voxel from high-resolution, intravital imaging data (Figure 1A and B, Video 1). This complex, spatial dataset was then reduced to a unique intensity distribution profile by normalizing all voxels within the 3D volume and plotting them as a percentage of total nuclear volume (Figure 1C). The normalization also accounts for any variation in raw intensity values among different nuclei, allowing for comparison among different cell populations and mice, irrespective of differences in mean intensity. Therefore, any changes in chromatin compaction profiles reflect the relative change in chromatin architecture intrinsic to the individual nucleus, that can therefore be compared to any other analyzed nuclei.

**Figure 1—figure supplement 1.**
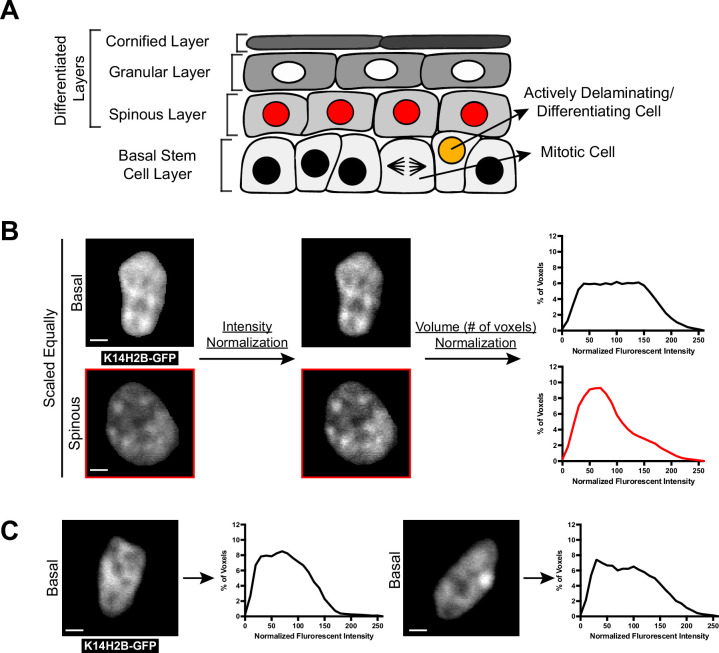
Chromatin compaction state analysis. (**A**) XZ schematic of the mouse epidermis. The basal stem cell layer (bottom) contains actively cycling cells as well as cells that have committed to differentiation and are actively leaving the layer. Cells differentiate apically until they eventually die and form the most outer barrier of the epidermis. (**B**) Quantitative workflow of the chromatin compaction analysis from imaging data (methods). Individual nuclei (left) are surfaced in 3D, exported as raw voxel data, normalized for mean intensity and plotted as a voxel percentage of total nuclear volume. These normalized voxels can then be exported back as scaled, intensity-based images (right). (**C**) Two individual basal nuclei are shown with their corresponding chromatin compaction histograms. All scale bars = 2 μm.

**Figure 1—figure supplement 2.**
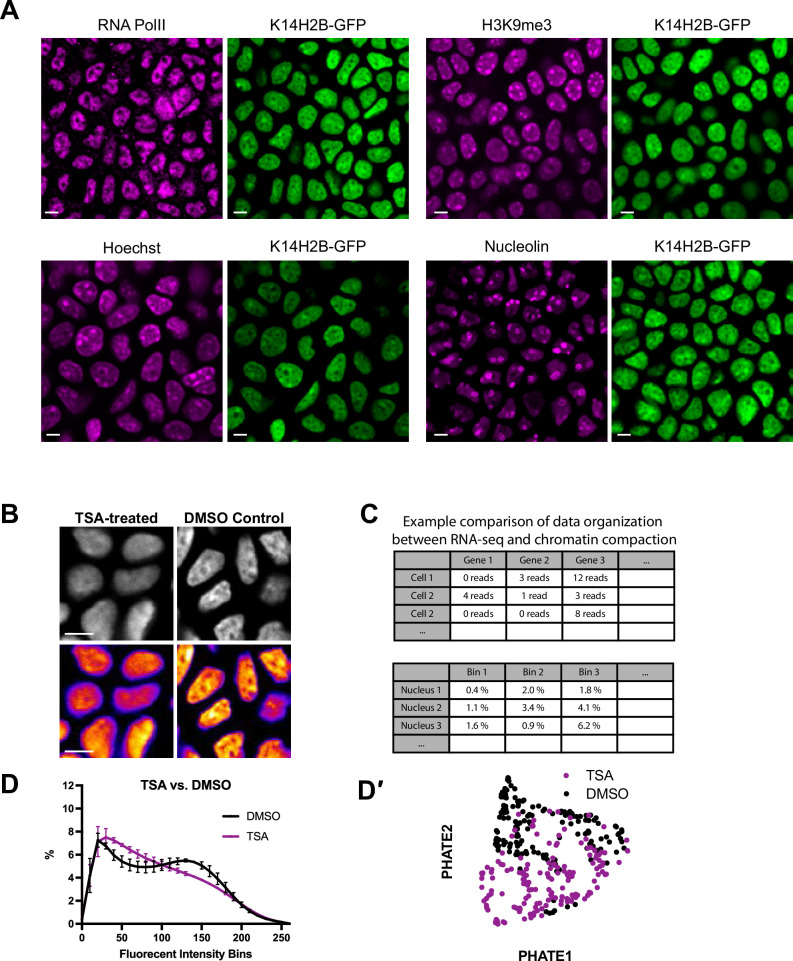
Organization of H2B-GFP allele. (**A**) Representative crops of fixed tissue, single channel stainings of the basal stem cell layer. Genetically encoded K14H2B-GFP signal is shown in green, and immunofluorescent stainings in magenta. Single optical slice. (**B**) Representative regions of TSA-treated (left) and DMSO vehicle control (right) mice showing clear disruption of chromatin distribution. K14H2B-GFP intensity seen in greyscale (top) and in the FIRE LUT (bottom). (**C**) Comparison of mock data between scRNA-sequencing data typically applied to the PHATE/Louvain clustering algorithm, and the imaging-based voxel intensity data used. (**D**) Chromatin compaction analysis for TSA-treated and DMSO control nuclei showing altered chromatin compaction state in the TSA-treated mice. N=150 basal stem cell layer nuclei across three mice. (**D′**) PHATE representation of data from (**E**) showing largely separated clusters of TSA and DMSO-treated mice. All scale bars = 5 μm. Mean and standard deviation among mice shown in panel D.

**Video 1.** Chromatin compaction. A single basal stem cell layer nucleus crop visualizing H2B-GFP intensity. The video first scans through the greyscale, intensity image (white), then through the FIRE LUT intensities shown in Figure 1B, then the three different binned intensities seen in Figure 1B and C. Nuclear outline is denoted by the white dotted line.

Applying this pipeline to intravital imaging data allowed us to quantify chromatin compaction within individual nuclei, among a population of stem cells, and between distinct cell identities based on cell location within the skin of a live mouse (Figure 1—figure supplement 1B and C).

The variable intensity of H2B-GFP indicates regions with different levels of chromatin compaction: highly-compressed, constitutively repressed chromocenters, loosely packed euchromatin, and nuclear periphery and nucleoli (Figure 1C). In fixed tissue staining, we recapitulated these chromatin compaction regions with Hoechst, as well as localization of known subnuclear compartments such as H3K9me3-positive chromocenters in the high H2B-GFP intensity regions, active RNA polymerase in the lower H2B-GFP intensity regions, and nucleoli in the very low H2B-GFP intensity regions (Figure 1D, Figure 1—figure supplement 2A). Applying the histone deacetylase inhibitor Trichostatin-A (TSA) abrogated these differences in H2B-GFP fluorescent intensity throughout individual nuclei and resulted in a shifted chromatin compaction curve compared to that of epidermal cells in the stem cell layer treated with the DMSO vehicle control (Figure 1—figure supplement 2B and D). Thus, the relative H2B-GFP intensity provides a visual readout of large-scale chromatin architecture (Figure 1C) and can be quantified to visualize the relative distribution of chromatin at different levels of compaction within individual nuclei irrespective of their volumes and mean H2B-GFP fluorescence intensities (Figure 1B).

Applying our chromatin compaction analysis to the basal stem cell layer, we noticed a clear heterogeneity among cells (Figure 1E). Previous studies have demonstrated chromatin organization heterogeneity at the level of individual locus accessibility or TAD boundaries (Buenrostro et al., 2015; Finn et al., 2019; Jin et al., 2015; Lai et al., 2018), but given that our chromatin compaction analysis captures very large-scale aspects of global chromatin architecture, the degree of heterogeneity we observed in the basal stem cell layer was surprising. Cells within the stem cell layer are in a spread of cell cycle states at any given time, so we hypothesized that cells may differentially condense their chromatin throughout the interphase cell cycle, producing an overall heterogeneity in chromatin compaction states reflective of cell cycle status. At any given point,~20% of the cells within the basal layer are in S/G2/M, and the remaining ~80% are in G1 (Hiratsuka et al., 2015). By inducing overexpression of the cell cycle inhibitor *p27* (*Cdkn1b*), we stalled the entire basal stem cell population in G1 (K14rtTA; tetO-Cdkn1b; K14H2B-GFP) (Figure 1F). Intriguingly, reducing cell cycle heterogeneity did not significantly change the population-level chromatin compaction state of basal stem cells (Figure 1G and H). To explore more subtle relationships between individual cells’ chromatin compactions states, we used the dimensionality-reducing data visualization algorithm PHATE (Moon et al., 2019). In this method, individual data points represent single cells, and the distance between points reflects the similarity of those cells’ chromatin compaction profiles. In our data, the proportion of each nucleus’ H2B-GFP intensity in each bin is analogous to each cell’s number of reads for each gene in the more typical single cell RNA-sequencing application of PHATE (Figure 1—figure supplement 2C). Plotting the combined wild-type and mutant populations together revealed that they largely intermix both within the homeostatic cell cycle distribution (day 0) and through the cell cycle stall in G1 (day 1) (Figure 1G′ and H′), which is in contrast to the largely separated populations of cells treated with TSA vs. DMSO (Figure 1—figure supplement 2D′). These data are consistent with previous studies which showed heterogeneity in chromatin architecture and accessibility independent of cell cycle differences (Buenrostro et al., 2015; Buenrostro et al., 2015; Finn et al., 2019; Lai et al., 2018). Together, these results show that single cell chromatin compaction states in the basal stem cell layer are heterogeneous and independent of interphase cell cycle status.

### Differentiation status of epidermal cells dictates chromatin compaction state

**Figure 2.**
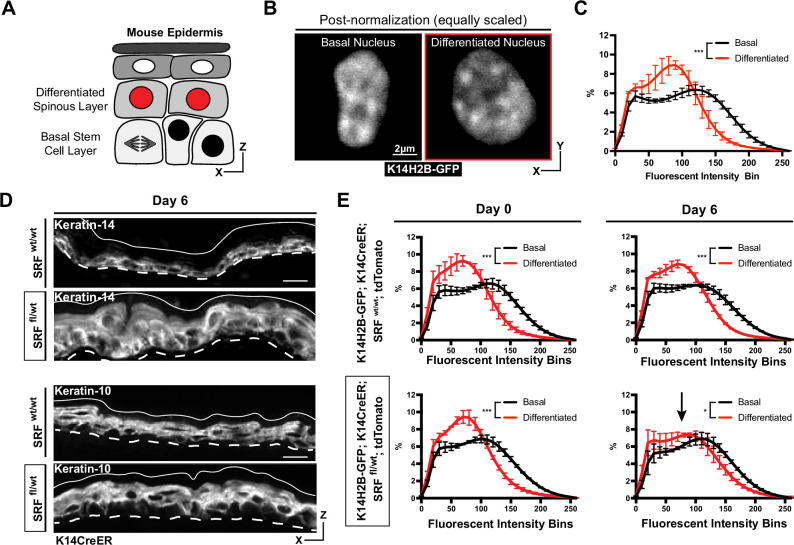
Chromatin compaction state changes through differentiation state. (**A**) XZ schematic of the epidermis. The basal stem cell layer is shown with black nuclei and the differentiated (spinous) layer is shown apical with red nuclei. (**B**) Representative crops of individual nuclei from the basal and differentiated populations scaled identically. H2B-GFP signal in white. (**C**) Chromatin compaction profiles of averaged populations of cells from the basal stem cell and differentiated layers showing significant differences in chromatin plots. N=150 basal and 90 spinous cells across 3 mice. (**D**) Fixed, XZ tissue slices from *Srf ^wt/wt^* and *Srf ^fl/wt^* mice day 6 after tamoxifen recombination. The basal stem cell marker, KRT14, can be seen expanded into differentiated layers, and the differentiated marker, KRT10, can be seen localized correctly despite a thickened overall epidermis. (**E**) Chromatin compaction profiles for wild-type (*Srf ^wt/wt^*; K14CreER; K14H2B-GFP; *R26^LSL-tdTomato^*) and mutant (*Srf ^fl/wt^*; K14CreER; K14H2B-GFP; *R26^LSL-tdTomato^*) mice on day 0 and 6 after tamoxifen recombination. Black arrow in *Srf ^fl/wt^* day 6 denotes that significant change in spinous cell chromatin compaction profile. N=150 basal and 90 spinous cells across 3 mice per day. Mean and standard deviation among mice shown in panels C and E. Figure 2—source data 1.Chromatin compaction state changes with differentiation status.

The epidermis is a highly regenerative organ involving the continual differentiation of cells from the basal stem cell layer to form the functional barrier of our skin. Cells within the stem cell layer will continually alter their transcriptome, delaminate from the stem cell compartment, and move apically/outward to form the overlaying differentiated layer (called spinous) over the course of 3–4 days (Cockburn et al., 2022; Mesa et al., 2018; Figure 2A). Previous studies have demonstrated that basal stem cells and differentiated spinous cells have different nuclear volumes and numbers of pericentromeric clusters (Gdula et al., 2013). With live imaging of H2B-GFP, we observe additional qualitative differences in chromatin architecture between these two populations; for example, differentiated spinous cells have a higher number of compact, bright chromocenters, and overall flatter nuclei (Figure 2B). By applying our chromatin compaction analysis to these two different cell populations, we observed that they have distinct chromatin compaction profiles, where the compaction state of differentiated cells was shifted toward lower fluorescent intensity bins (Figure 2C). As the quantitative pipeline allows for comparison between nuclei irrespective of differences in mean fluorescent intensity values, the shift left for differentiated cells implies a relative increase in very low-intensity regions such as the nuclear lamina and nucleoli, with a relative decrease in euchromatic and heterochromatin regions (Figure 1—figure supplement 1B).

**Figure 2—figure supplement 1.**
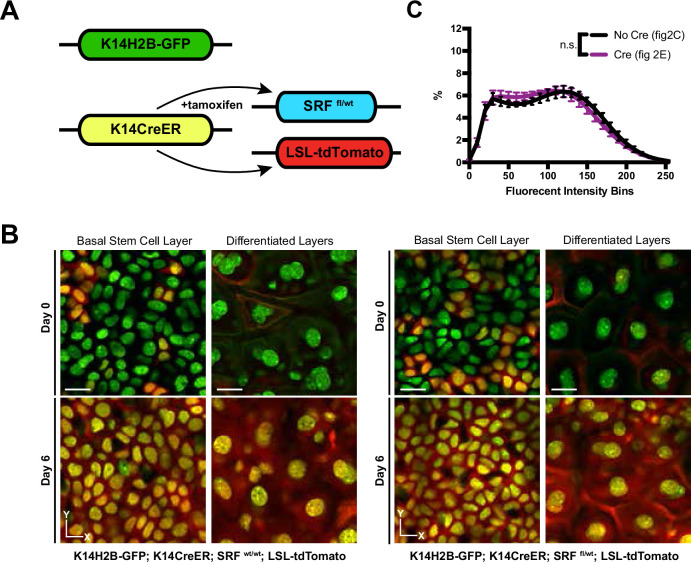
Genetic ablation of SRF alle in the basal stem cell layer. (**A**) Schematic of the inducible genetic system used in Figure 2 to recombine and knock down *Srf*. (**B**) Representative crops from the basal stem cell and differentiated layers on days 0 and 6 in wild-type (top) and *Srf* heterozygous (bottom) mice. *R26^LSLtdTomato^* acts as a readout of recombination. (**C**) Chromatin compaction plots from Figure 2C and E (upper right panel) to determine the effect of Cre expression on chromatin compaction measurement. All scale bars = 10 μm. Mean and standard deviation among mice shown in panel C.

To better understand how differentiation affects chromatin compaction state, we genetically knocked out one copy of *Serum Response Factor* (*Srf*) in the epidermis of adult mice (K14H2B-GFP*; Srf ^fl/+^;* K14CreER*; R26^LSL-tdTomato^*) (Figure 2—figure supplement 1A). SRF is a transcription factor which helps establish proper cell identity in the epidermis, contributing both to embryonic skin stratification and proper stem cell differentiation in the adult mouse epidermis (Lin et al., 2013; Verdoni et al., 2010). At 6 days post-recombination, the loss of SRF caused a transcriptional identity shift in the skin; expression of the basal stem cell marker Keratin-14 was no longer restricted to the basal stem cell layer, despite grossly normal epidermis organization (Figure 2D). Notably, expression of Cre did not in itself change chromatin compaction of epidermal cells (Figure 2—figure supplement 1C). In both SRF heterozygous and wild type control mice, the chromatin compaction states in basal and spinous cells were distinct on day 0. By day 6, the differentiated, spinous population’s chromatin compaction was more basal-like in the SRF heterozygous mice, while the WT controls maintained distinct chromatin compaction profiles between the cell identities (Figure 2E). In particular, the chromatin compaction state of spinous differentiated cells shifted towards the chromatin compaction state of basal stem cells, reflecting the expansion of Keratin-14 expression into the spinous layer and the breakdown of transcriptome identity between the two cell populations. Importantly, these data also demonstrate that chromatin compaction changes can be reflective of changes in transcriptional program and cell identity, even prior to extensive tissue phenotypes.

### Basal cell chromatin compaction is stable over hours, but transitions through differentiation over days

**Figure 3.**
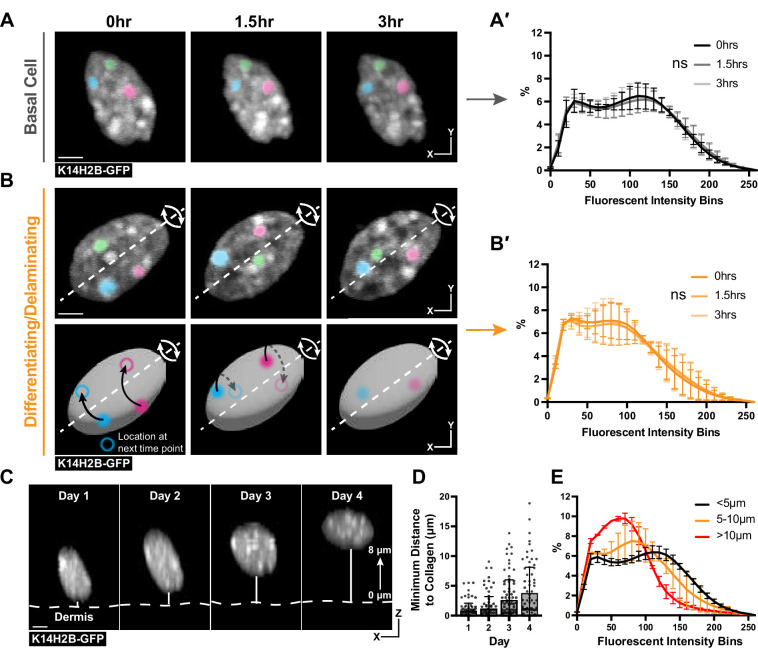
Chromatin compaction is stable over hours and progressively changes over days. (**A**) Time lapse imaging data of a single nucleus crop at 0, 1.5, and 3 hr time points. H2B-GFP fluorescent signal is shown in white. Three, high-intensity chromocenters were chosen and pseudo-colored blue, green, and pink to demonstrate their static nature over the 3 hr. Max projection. (**A′**) Chromatin compaction profiles for cells in the basal stem cell layer at the 0, 1.5, and 3 hr time points showing no significant change over 3 hr. N=150 basal cells across 3 mice. Scale bar = 2 μm. (**B**) Same as in (**A**) but cells actively delaminating out of the basal stem cell layer, exhibiting rolling nuclei. The axis of rotation is show in the white dotted line, with the rotational direction shown in white arrows around that axis. Three, high-intensity chromocenters were chosen and pseudo-colored blue, green, and pink to demonstrate the dynamic spinning taking place, but the positional stability of global chromatin organization relative to itself. A cartoon (below) of the same nucleus to better visualize the rotation and orientation over the 3 hr with the blue and pink pseudocolored chromocenters tracked through time. Black arrows indicate where the chromocenter will move to in the next time point (hollow circle), dotted black arrow indicates rotation around the backside of the nucleus. Scale bar = 2 μm. Max projection. (**B′**) Chromatin compaction profiles for cells with spinning chromatin (actively delaminating cells) showing no significant change over 3 hr. N=146 spinning/delaminating cells over 3 mice. (**C**) XZ crops of the same nucleus tracked within the tissue over 4 days. Representative example of a differentiating cell over this time period. Bold, dotted white line denotes the epidermal/dermal interface. Solid, thin white line shows the minimum distance from collagen quantified in panel D. Scale bar = 2 μm. (**D**) H2B-GFP fluorescent signal shown in white. (**D**) Minimum distance from collagen for a randomly selected population of basal cells on day 1, some of which differentiated and moved apically by day 4, while others remained basally located. (**E**) Chromatin compaction profiles of nuclei within the tracked population from panel (D) binned as distance from collagen showing the direct transition in chromatin compaction profiles through differentiation. N=150 basal stem cells tracked over 4 days from 3 mice. Mean and standard deviation among mice shown for all chromatin compaction profiles. Figure 3—source data 1.Chromatin compaction state changes slowly over days.

Because we observed that chromatin is differentially compacted and organized between the basal stem cell and differentiated (spinous) layer above, we hypothesized that chromatin architecture would reorganize at a specific transition point during stem cell differentiation. To test this hypothesis, we began by analyzing chromatin compaction from 3-hr timelapse imaging of K14H2B-GFP mice. Visually, chromatin compaction in cells in the basal stem cell layer was relatively stable over this time. High H2B-GFP density regions appeared to move only slightly, and chromatin compaction profiles at the beginning, middle, and end of these 3-hr time lapses exhibited no significant change at the individual cell or population level (Figure 3A and A′, Figure 3—figure supplement 1A). These findings demonstrated that among these heterogeneous basal stem cells, the chromatin compaction state of each cell is stable over hours.

**Figure 3—figure supplement 1.**
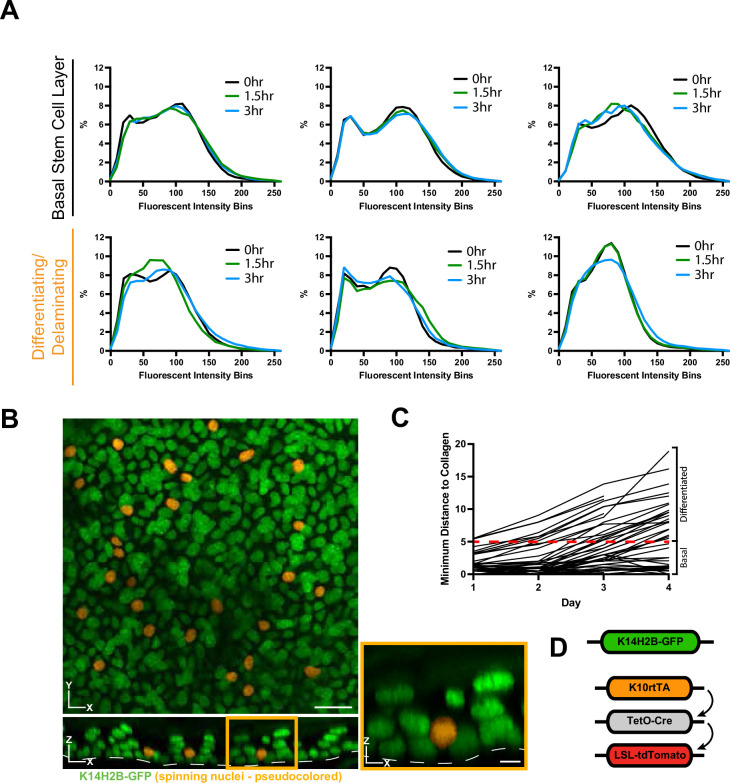
Single-cell chromatin compaction dynamics over hours. (**A**) Individual nucleus chromatin compaction profiles from 3 basal stem cell and 3 delaminating/spinning cells at 0-, 1.5-, and 3 hr time points showing little change over this time scale. (**B**) Representative XY (top) and XZ (bottom) max projection FOV of the epidermis (K14H2B-GFP in green) with spinning chromatin pseudocolored in orange. (**C**) Individual tracking of cells from Figure 3D showing trajectory of minimum distance from collagen over 4 days. (**D**) Schematic of the inducible genetic system used in Figure 4 to visualize cells within the basal stem cell layer that have started expressing *Keratin-10*. Scale bar (left)=15 μm. Scale bar (inset)=5 μm.

We next wondered whether the transition in chromatin compaction occurs slightly later during differentiation in cells actively delaminating, located in between the basal and spinous layer (Cockburn et al., 2022; Figure 3—figure supplement 1B). Strikingly, we noticed the chromatin of these cells spin over the course of the timelapse (Figure 3B, Video 2). The H2B-GFP allele allows us to only visualize spinning of the chromatin, but it is likely that the entire nucleus of these cells is rotating, based on observations of nuclear spinning in other systems (Kumar et al., 2014; Zhu et al., 2018). The chromatin spinning was observed in all timelapses obtained under homeostatic conditions. Surprisingly, even as the chromatin as a whole spun, there were no significant changes in chromatin compaction (Figure 3B′). Notably, though, the chromatin compaction state of these actively delaminating cells was markedly different from the chromatin compaction state of basal cells (compare **3B′** to **3 A′**), and appeared to be an intermediate differentiation state between basal and spinous cells. Overall, these timelapse data suggest the transition in chromatin compaction state during epidermal differentiation occurs over days, not hours, and is already in progress during basal cell delamination.

**Video 2.** Spinning chromatin. An XY field-of-view of the upper, basal stem cell layer in which chromatin (H2B-GFP) can be observed to spin. Timelapse is 3 hours long and looped 3 times.

To test this hypothesis, we tracked a population of cells in the basal stem cell layer over four days in a live mouse (Figure 3C). By doing so, we sought to understand when and how quickly chromatin compaction changes were taking place, as well as confirming that the cells progressively transition between the chromatin states seen in Figure 2B and C. This population increased their average minimum distance from collagen from day 1 to day 4, reflecting that a portion of these cells were differentiating and moving apically into the spinous layer of the epidermis (Figure 3C and D, Figure 3—figure supplement 1C). After tracking all cells over this time period, we binned nuclei into groups determined by their minimum distance to collagen to quantitatively measure the delamination process. When the chromatin compaction analysis was applied to these populations, we observed a gradual and directional change in chromatin compaction state as cells moved farther from collagen and moved into the spinous layer (Figure 3E). Together, our results indicate that chromatin compaction remodels slowly over days concomitant with differentiation and delamination.

### Chromatin compaction state begins to transition within the basal stem cell layer as differentiation initiates

The observation that delaminating basal cells had chromatin compaction states similar to differentiated spinous cells (Figure 3B–B**′**) made us wonder whether chromatin reorganization began while cells were still within the basal stem cell layer. This model could also explain the heterogeneity of chromatin compaction states within the basal layer (Figure 1E). Indeed, previous studies have shown that basal stem cell differentiation involves cumulative transcriptional changes that begin prior to delamination (Aragona et al., 2020; Cockburn et al., 2022). We wondered if the chromatin compaction state of basal cells that are committed to differentiation differs from basal cells that are not.

**Figure 4.**
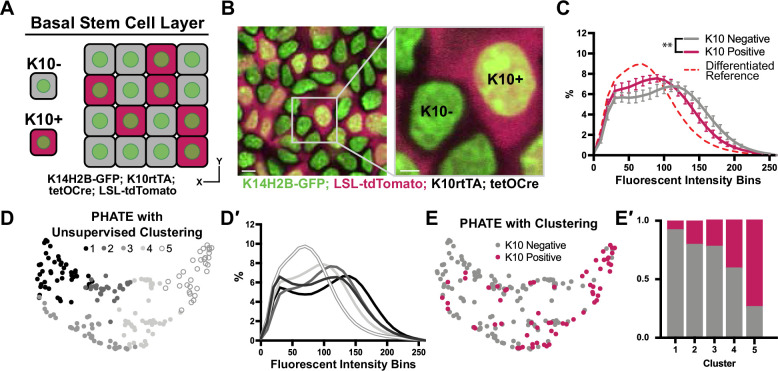
Chromatin compaction changes precede differentiation. (**A**) XY schematic of genetic system (K14H2B-GFP; K10rtTA; tetO-Cre; *R26^LSL-tdTomato^*) allowing visualization of actively differentiating cells still within the basal stem cell layer (expressing differentiation-associated *Keratin-10* gene). *Keratin-10*-positive cells indicated with red cytosol. (**B**) Representative XY crop of the basal stem cell layer showing *Keratin-10*-negative (no tdTomato signal) and *Keratin-10*-positive cells (tdTomato in cytosol) with a cropped inset on the right. Scale bar (left)=5 μm. Scale bar (inset)=2 μm. Max projection of basal stem cell layer. (**C**) Chromatin compaction profiles comparing *K10* status (tdTomato on/off) in basal stem cells showing significant differences in chromatin compaction between groups. A differentiated reference line is shown by the dotted, red curve. Averaged *K10* + cells shown in pink and averaged *K10*- cells shown in grey. N=187 *K10-* nuclei and N=132 *K10*+ nuclei from 3 mice. Statistical comparisons made between histogram groups, p<0.01. (**D**) PHATE plot of data from (**C**). Louvain clustering results are projected onto the PHATE plot. Each dot represents one nucleus profile, and the distance between dots represents the similarity in chromatin compaction profile. (**D′**) Chromatin compaction profiles of the averaged clusters identified through Louvain clustering in (**D**) elucidating directionality in the clustering from a more basal curve to a more differentiated curve. (**E**) The same PHATE/clustering dataset as in (**D**) with overlayed *K10* status (on/off) again demonstrating directionality in the PHATE map. (**E′**) The ratio of *K10* positive (red) and negative (grey) in each of the Louvain clusters. Mean and standard deviation among mice shown in panel C. Figure 4—source data 1.Chromatin compaction state begins remodeling upstream of basal delamination.

To test this hypothesis, we genetically labelled the cells of the basal layer with an early marker of differentiation: expression of *Keratin-10* (K10rtTA; tetO-Cre*; R26^LSL-tdTomato^;* K14H2B-GFP; Cockburn et al., 2022; Muroyama and Lechler, 2017; Figure 4A and B, Figure 3—figure supplement 1D). This population represents a relatively large portion of the basal stem cell layer (~40%), and expression of the *K10* reporter has been shown to define a point of commitment for delamination (Cockburn et al., 2022). Basal cells were sampled with respect to *K10* reporter (tdTomato) expression, and then binned into *K10+* and *K10-* groups. Remarkably, even this relatively small difference in cell states (all a part of the basal stem cell layer) had significantly different chromatin compaction profiles (Figure 4C). In addition, the cells positive for the *K10* reporter adopted an intermediate chromatin compaction profile which was shifted towards that of the fully differentiated cells in Figure 2C and delaminating/differentiating cells in Figure 3B′. These data highlight that basal stem cells initiate global chromatin changes coinciding with delamination and exit from the stem cell compartment.

Applying the PHATE analysis and an unsupervised Louvain clustering algorithm to this basal stem cell dataset (including both *K10-* and *K10+* cells), five distinct clusters emerged (Figure 4D). The chromatin compaction profiles of the cells in the five clusters showed an entire spread of curves from most ‘basal-like’ (cluster 1) to nearly fully differentiated (cluster 5) (Figure 4D′), reflecting the overall differentiation trajectory of chromatin compaction states. Together with the shape of the PHATE map itself, we noticed that the left and right sides (clusters 1 and 5) of the map seemed to narrow and pinch together, implying more similar chromatin compaction states among cells at the beginning and end of this trajectory than among the cells in the middle of the trajectory (clusters 2–4) (Figure 4D). Because these maps were derived from the *K10* reporter dataset, we were able to overlay each cell’s *K10* status onto the PHATE maps (Figure 4E). The distribution of *K10*+ and *K10*- cells within the PHATE map clusters (Figure 4E′) reinforced the directionality of chromatin compaction changes indicated in Figure 4D′. Together, these data show that changes in chromatin compaction coincides with stem cell commitment to differentiation.

### Dynamic *Keratin-10* expression precedes global chromatin compaction changes in differentiating basal stem cells

**Figure 5.**
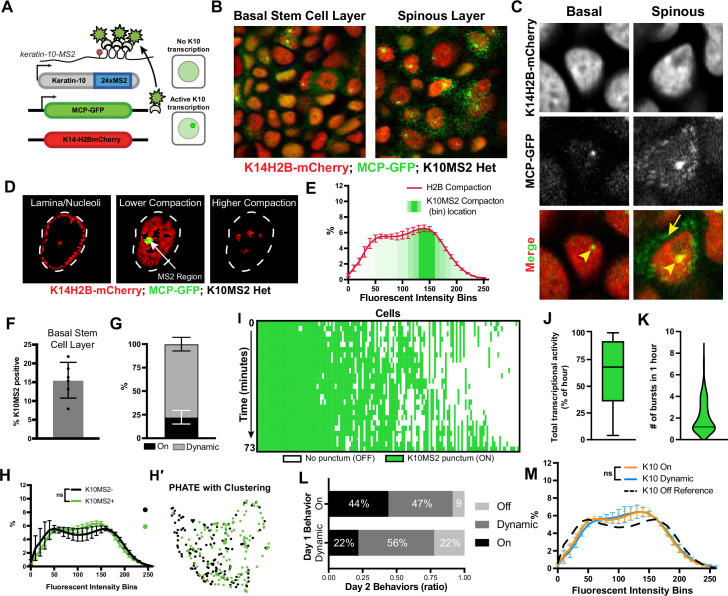
in vivo transcription of *Keratin-10* precedes genome architecture changes through differentiation. (**A**) Visual schematic of the MCP/MS2 system allowing visualization of a targeted gene under endogenous regulation. 24X MS2 repeats were knocked into the 3′UTR of the *Keratin-10* locus and chromatin compaction visualized with K14H2B-mCherry. Presence of nuclear MCP punctum indicates active *Keratin-10* transcription, where lack of signal indicates no active transcription at that locus. (**B**) Representative field of view of the *K10*MS2(het)/MCP-GFP system in the basal (left) and spinous (right) layers. Nuclei labeled in red and *Keratin-10* RNA in green. Sum projection through each layer. (**C**) High-resolution insets of the *K10*MS2(het)/MCP-GFP system in basal and spinous cells. Yellow arrowhead = site of active *Keratin-10* transcription inside the nucleus. Yellow arrow = mature *Keratin-10* transcripts in the perinuclear space. Sum projection through nuclei. (**D**) Representative image of a nucleus separated into the same chromatin compaction regions as in Figure 1C. Active transcription of *Keratin-10* can be seen within the lower compaction region from bins 100–200. Scale bar = 2 μm. Single optical slice. (**E**) Chromatin compaction profile of averaged H2B-mCherry basal stem cells (red line) and the fluorescent bin location of *Keratin-10* transcription punctum (green, semi-translucent bars). N=150 *Keratin-10* transcribing basal stem cells across 3 mice. Surfaced MCP signal was used to identify the mCherry fluorescent intensity bins in which *Keratin-10* transcription occurred, and then plotted as increasing green transparency. (**F**) Populational percentage of active *Keratin-10* transcription in the basal stem cell layer in *K10*MS2 Het mice. N=3 100 x 100 μm regions quantified over 3 mice. (**G**) Percentages of *Keratin-10* dynamics within the *Kertain-10*-positive basal stem cell layer over 1 hour. ~25% of basal stem cells actively expressing *Keratin-10* remained on throughout the 1 hr timelapse, while ~75% had dynamic transcriptional behaviors. (**H**) Chromatin compaction analysis of basal stem cells either actively transcribing *Keratin-10* (green punctum in nucleus) or not transcribing *Keratin-10*. Despite active transcription of the differentiation gene, there is no significant chromatin compaction remodeling at this stage. N=150 *Keratin-10* ‘off’ and 150 ‘on’ nuclei across 3 mice. (**H′**) PHATE plot of the data in (**I**) showing intermixed populations of *Keratin-10* positive and negative basal stem cells. (**I**) *Keratin-10* dynamics over 1.25 hr of cells that actively transcribed *Keratin-10* for at least one time point. Cells ordered by total amount of time transcribing *Keratin-10* over the timecourse. N=148 over 3 mice. (**J**) Average *Keratin-10* total transcriptional activity over 1.25 hr per cell. (**K**) The number of *Keratin-10* bursting events over 1.25 hours per cell. (**L**) Quantification of how *Keratin-10* dynamics change over the course of 1 day. *Keratin-10*-positive nuclei from day 1 were binned into ‘on’ and ‘dynamic’ (y-axis) and the same nuclei located on day 2. Day 2 transcriptional dynamics were quantified (x-axis) showing surprising flexibility in transcriptional dynamics over a day. (**M**) Chromatin compaction analysis of the ‘on’ and ‘dynamic’ *Keratin-10* transcription populations from (**F**) showing very little, non-significant differences between the two populations. The *Keratin-10* ‘off’ curve from (**H**) is shown as a reference in the dotted, black line. N=187 ‘dynamic’ and 124 ‘on’ over 3 mice. Mean and standard deviation among mice shown for all chromatin compaction profiles. Figure 5—source data 1.Transcription of differentiation gene is highly dynamic and precedes significant chromatin compaction changes.

Intrigued by how the differentiation trajectory seemed to be reflected in chromatin compaction states, we wanted to more specifically understand the relationship between global chromatin compaction state and transcription at the *Keratin-10* locus. To do so, we used the virally-derived MS2/MCP genetic system to visualize *Keratin-10* transcripts in real time. We knocked in the MS2 cassette (24 x repeats of the MS2 stem loop sequence) after the stop codon of the *Keratin-10* locus, and then crossed this mouse line to the MCP-GFP reporter mouse (Lionnet et al., 2011) and *TIGRE*-K14-H2B-mCherry (Methods). MCP-GFP binds the MS2 stem loops, and this happens as soon as *Keratin-10-MS2* is transcribed. Thus, these mice (*K10*MS2*/+;* MCP-GFP/+*; TIGRE*-K14-H2B-mCherry) allowed us to visualize not only chromatin architecture through mCherry-tagged H2B, but also in vivo transcription of an endogenous allele through a GFP-positive punctum in the nucleus (Figure 5A, Figure 5—figure supplement 1A). Note that only one punctum is visible because these mice were heterozygous for the *K10-*MS2 allele. Importantly, while both the *K10-*MS2/MCP-GFP transcription reporter and the K10rtTA reporter in Figure 4 will capture cells that have been stably expressing *Keratin-10* for some time, the transcription reporter can capture cells at an earlier stage of *Keratin-10* expression compared to the K10rtTA reporter, which relies on multiple successive steps of gene expression, translation, and recombination.

**Figure 5—figure supplement 1.**
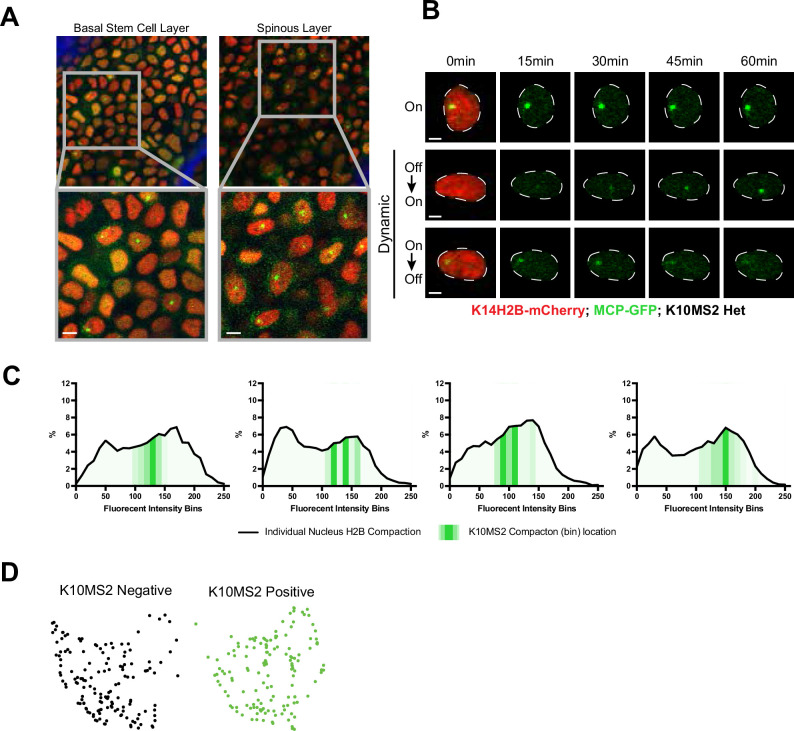
Imaging differentiation-associated cell identity. (**A**) Representative crops of the differentiated spinous layer (left) and basal stem cell layer (right), as well as magnified inset showing active site of *Keratin-10* transcription, and nascent transcripts in cytosol awaiting translation. Scale bar = 5 μm. (**B**) Representative crops of individual basal stem cell nuclei exhibiting different *Keratin-10* transcriptional dynamics over the course of 1 hr. H2B-mCherry signal can be seen in red in the first time point for reference, and *Keratin-10* transcription punctum in green in all time points. White, dotted line shows the nuclear border. Scale bar = 2 μm. (**C**) Individual nuclear chromatin compaction profiles for four basal stem cell layer nuclei. K14H2B-mCherry compaction profile shown by black line, and the MCP/MS2 *Keratin-10* transcription regions in green translucency. (**D**) PHATE representation from Figure 5H′ separated by cell status (*Keratin-10* ‘on’ or ‘off’). While largely intermixed, there appears a slight preference (though non-significant) for one side of the PHATE cluster vs. the other (‘off’ to the left, ‘on’ to the right).

Imaging the epidermis of these mice confirmed that differentiated spinous cells had active transcription of *Keratin-10*, and only a subset of cells in the basal stem cell layer had active *Keratin-10* transcription at any given time (Figure 5B and C), which is consistent with previous characterizations of Keratin-10 protein expression patterns (Braun et al., 2003; Doupé et al., 2010; Schweizer et al., 1984). To further validate that MCP-GFP puncta represent active *Keratin-10* transcription at its locus, we next sought to resolve the local chromatin environment at the *Keratin-10* locus during active transcription. To do so, all H2B-mCherry fluorescence voxels within the nuclear surface of MCP-GFP puncta-containing basal stem cells were normalized as previously described, and those voxels that overlapped with the surface of MCP-GFP signal were extracted, enabling a quantitative visualization of the H2B-mCherry fluorescence within the local chromatin environment of *Keratin-10* transcription. This analysis revealed that *Keratin-10* transcription primarily occurred within loosely packed euchromatic regions of the nucleus (Figure 5D and E, Figure 5—figure supplement 1C), as expected.

Interestingly, while 15% of basal cells had a strong MCP-GFP punctum in the nucleus at any given time (Figure 5F), indicating active transcription, we also observed cells with multiple, dimmer MCP-GFP puncta in the cytosol without active transcription of *Keratin-10* (no nuclear punctum) (Figure 5C). This latter group led us to believe transcription of *Keratin-10* might be quite dynamic, with kinetics in the range of the half-life of the *Keratin-10* mRNA itself, likely minutes to hours (Dar et al., 2012; Suter et al., 2011). To understand possible *Keratin-10* transcription dynamics, we iteratively imaged *Keratin10-*MS2/+; MCP-GFP/+; K14H2B-mCherry mice at roughly 3 min intervals over the course of approximately 1.25 hr. Intriguingly, of the cells actively transcribing *Keratin-10*, about 25% sustained *Keratin-10* transcription with no notable change in fluorescence intensity, but about 75% displayed dynamic transcription over the timelapse – they turned *Keratin-10* on or turned *Keratin-10* off during the hour imaged (Figure 5G and Figure 5—figure supplement 1B). By quantifying each cell’s *Keratin-10* transcriptional activity, we were able to uncover in vivo transcriptional dynamics such as burst duration and frequency over the timelapse (Figure 5I). The *K10*MS2 allele displayed a wide range in both total transcriptional activity and transcriptional burst duration, ranging from a single time step during imaging (3 min) to fully active for the duration of the time lapse (1.25 hr) (Figure 5I–J). Finally, basal cells actively transcribing *Keratin-10* ranged in a burst frequency of 1 (transcribing for the whole hour) to greater than 4 (Figure 5K). These data indicate that the fast transcriptional dynamics (transcriptional ‘bursting’) which have been thoroughly characterized in cell culture and other model organisms (Dar et al., 2012; Suter et al., 2011; Bothma et al., 2014; Chubb et al., 2006; Fritzsch et al., 2018) are mirrored for this mammalian differentiation gene in vivo.

To determine the perdurance of *Keratin-10* transcriptional dynamics, we performed timelapse imaging of the exact same cells in *Keratin10-*MS2/+; MCP-GFP/+; K14H2B-mCherry mice 24 hr apart. We observed that only a subset of cells displayed the same transcriptional behaviors between the 2 days – approximately 40% of *Keratin-10* ‘on’ cells were ‘on’ the next day, while roughly 60% *Keratin-10* ‘dynamic’ cells were ‘dynamic’ the next day. The remaining cells which had been transcribing *Keratin-10* on day 1 displayed a variety of different *Keratin-10* transcriptional behaviors on day 2, including an absence of active transcription entirely (Figure 5L). Collectively, these data indicate that *Keratin-10* transcriptional activity in basal stem cells is flexibly dynamic across days as well as minutes.

To understand how *Keratin-10* transcriptional status relates to chromatin compaction states, we used the H2B-mCherry signal to perform chromatin compaction analysis. This analysis revealed that cells actively transcribing *Keratin-10* had chromatin compaction states similar to that of cells not transcribing *Keratin-10* (Figure 5H and H′, Figure 5—figure supplement 1E), although they trended towards the chromatin compaction state of cells expressing the K10rtTA reporter (compare Figure 5H to Figure 4C). Because the cells marked by the *Keratin-10-*MS2/MCP-GFP transcription reporter include those at the very earliest step of differentiation, this result suggests that chromatin compaction changes either have not yet occurred or are just beginning to occur when *Keratin-10* transcription is initiated.

Finally, we hypothesized that *Keratin-10* ‘on’ cells were further advanced in their cell identity transition toward differentiation than *Keratin-10* ‘dynamic’ ones. Intriguingly, the chromatin compaction signature of *Keratin-10* ‘on’ compared to *Keratin-10* ‘dynamic’ cells were extremely close to one another (Figure 5M) suggesting that the cells displaying these different *Keratin-10* transcriptional dynamics in fact co-exist within extremely similar cell identity states.

Altogether, these results reveal that the initiation of *Keratin-10* transcription, which represents one of the first steps of a basal stem cell towards differentiation, is highly dynamic and that significant chromatin remodeling occurs after transcription initiation of *Keratin-10*.

## Discussion

**Figure 6.**
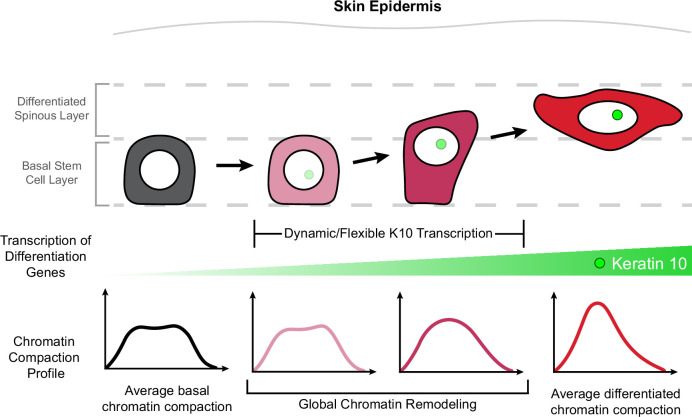
Chromatin architecture remodeling through epidermal differentiation. Epidermal stem cells undergo incremental changes toward differentiation over 3–4 days (top). During this process, differentiation-committed cells within the basal stem cell layer begin expressing *Keratin-10* dynamically over hours and flexibly over days (green circle/site of transcription in nucleus). Basal stem cell and fully differentiated spinous cells have specific chromatin architecture states associated with their different cellular identities (bottom row), and global chromatin remodeling begins before delamination and exit from the basal stem cell layer.

Homeostasis and function of regenerative tissues requires constant self-renewal and differentiation of resident stem cells. Stem cells undergo a relatively large reorganization of their genome through the differentiation process as their cell identity changes (Kurimoto et al., 2015; Oudelaar et al., 2020; Paulsen et al., 2019; Li et al., 2017). Current tools offer high-resolution data about the chromatin environment at specific loci, but rely on fixed cells and are unable to reveal how chromatin architecture is remodeled during cell identity changes such as differentiation. Here, through high-resolution imaging of H2B-GFP in live mice, we discovered chromatin compaction heterogeneity within the epidermal stem cell population under homeostatic equilibrium. This heterogeneity arises from gradual, incremental shifts in stem cell identity throughout differentiation which begin prior to exit from the stem cell layer. Moreover, by live imaging endogenous transcription of *Keratin-10,* a hallmark of epidermal differentiation, we resolved highly dynamic transcriptional activity over hours and days in the absence of significant chromatin architecture changes. Ultimately, we determined that most of the global genome reorganization associated with cell identity transitions occurs between the initiation of differentiation-associated transcription and exit from the stem cell layer (delamination; Figure 6**, model**).

Our ability to live image chromatin compaction in the same cells over hours and days allowed us to discover that the global chromatin compaction state of epidermal stem cells is stable over the course of hours, but remodels through the differentiation process over days. Recently, other studies which performed live imaging in cell culture systems to evaluate chromatin dynamics at finer scales, such as individual loci and Topologically Associated Domain (TAD) boundaries, have revealed that chromatin can be locally dynamic over minutes and seconds (Barth et al., 2020; Chen et al., 2018; Gabriele et al., 2022; Iida et al., 2022). These dynamic local changes to individual loci and TADs may act cumulatively toward a global and incremental shift in chromatin architecture through differentiation.

By visualizing chromatin compaction changes at the same time as morphological and transcriptional changes, our results add to a growing understanding that the first steps of epidermal stem cell differentiation are somewhat flexible. Recent work tracking epidermal cell fates over a week showed that differentiating basal cells expressing *Keratin-10* are still capable of proliferation (Cockburn et al., 2022), a behavior that was previously assumed to be unique to epidermal stem cells. Additionally, pseudo-time analysis of scRNA-sequencing data from the same study revealed a significant population of cells that contain both stem and differentiation-associated transcripts (*Keratin-14* and *Keratin-10,* respectively). While the dynamic nature of *Keratin-10* transcription we observed within a one-hour timelapse could reflect the fact that that many genes exhibit transcriptional bursting kinetics on the scale of minutes (Dar et al., 2012; Suter et al., 2011), the observation that a portion of cells actively transcribing *Keratin-10* no longer express it 1 day later supports the idea that individual cells remain somewhat flexible in their initial commitment to differentiation (Figure 5L). Understanding at what point individual cells irreversibly commit to differentiation and can no longer proliferate, as well as if chromatin compaction state is also flexible during differentiation, remain interesting questions for future studies.

We have begun to tease apart the intimate relationship between chromatin architecture changes and transcriptional behavior in vivo. By combining two different genetic approaches to temporally visualize differentiation state (Figure 4 and Figure 5), our results suggest that transcriptional changes happen either before or right at the beginning of global chromatin remodeling. Recent papers have supported the bi-directional and reciprocal nature of chromatin organization at individual gene loci and transcription of those loci (Lai et al., 2018; Oudelaar et al., 2020; Li et al., 2021). Through our live imaging approach, we have greatly enriched our understanding of the interplay between the highly dynamic nature of transcription and local chromatin architecture by tracking cell identity transitions in a live mammal.

Finally, this chromatin compaction system is not dependent on biology inherent to the skin or epidermis, and in fact is widely applicable to other systems due to the use of H2B-GFP in many model systems. Additionally, this approach could be used to investigate other biological transitions in cell identity such as oncogenic initiation and expansion, and mesenchymal transitions through wound healing. There is also an opportunity to combine this more global view of chromatin architecture with higher resolution imaging modalities of individual loci or TAD boundaries. More broadly, these findings open a door into tissue-level coordination and flexibility among cells, and how the incremental and stepwise journey through differentiation establish heterogeneous cell states. We believe taking a more global view of these individual cell states, such as this tracking of pan-histone labeling, is one avenue to understand such processes.

## Methods

### In vivo imaging

All imaging was performed in non-cycling regions of the ear skin with hair removed using depilatory cream (Nair) before the start of each experiment. Mice were anesthetized using 1–2% vaporized isoflurane delivered by a nose cone throughout the course of imaging. Image stacks were acquired with a LaVision TriM Scope II (LaVision Biotec, Germany) laser scanning microscope equipped with both a Chameleon Vision II and Discovery 2-photon lasers (Coherent, USA). For collection of serial optical sections, the laser beam was focused through a 40 x water immersion lens (Nikon; N.A. 1.15) and scanned with a field of view of 200x200 um at 600 Hz. Z-stacks were acquired with 0.5–1 μm steps to image a total depth of ~40 μm of tissue, covering the entire thickness of the epidermis. Visualization of ECM was achieved via second harmonic signal using blue channel at 940 nm imaging wavelength. To follow the same epidermal cells over multiple days, inherent landmarks of the skin together with a micro-tattoo were used to navigate back to the same epidermal regions every 24 hr (Pineda et al., 2015). For time-lapse imaging, serial optical sections were obtained in a range of 15–30 min intervals for a total duration of 1–3 hr.

### Immunofluorescence

For SRF tissue-section analysis, ear skin was dissected, fixed with 4% paraformaldehyde in PBS for 1 hr at room temperature and then embedded in optimal cutting temperature (OCT; Tissue Tek). Frozen OCT blocks were sectioned at 10 μm. Primary antibodies used were guinea pig anti-K10 (1:200; Progen PG-K10) and rabbit anti-K14 (1:200; BioLegend 905301). All secondary antibodies used were raised in a donkey host and were conjugated to AlexaFluor 568 or 633 (Thermofisher). Fixed tissue was mounted on a slide with Vectashield Anti-fade mounting medium (Vector Laboratories) with a #1.5 coverslip.

To isolate epidermis for whole mount staining, ear tissue was incubated in 5 mg/ml dispase II solution (Sigma, 4942078001) at 37 °C for 10 min and the epidermis was separated from dermis using forceps. Epidermal tissue was fixed in 4% paraformaldehyde in PBS for 45 min at room temperature, washed 3 X in PBS, permeabilized and blocked for >1 hr (2% Triton-X, 5% Normal Donkey Serum, 1% BSA in PBS), incubated in primary antibody overnight at 4 °C, and secondary antibodies for 3 hr at room temperature the next morning. Primary antibodies used were as follows: guinea pig anti-K10 (1:200; Progen GP-K10), rabbit anti-K14 (1:200; BioLegend 905301) rabbit anti-H3K9me3 (1:200; Abcam ab8898), rabbit anti-nucleolin (1:200; Abcam ab22758), and rabbit anti RNA Polymerase pS2 (1:500; Abcam ab5095). All secondary antibodies used were raised in a donkey host and were conjugated to AlexaFluor 568 or 633 (Thermofisher). Fixed tissue was mounted on a slide with Vectashield Anti-fade mounting medium (Vector Laboratories) with a #1.5 coverslip.

### Image analysis

Raw image stacks were imported into FIJI (ImageJ, NIH) or Imaris (Bitplane) for analysis. Individual optical planes or max Z-stacks of sequential optical sections were used to assemble figures. Identification of the basal stem cell and differentiated layers/cells was determined with immunofluorescent staining, positional location within the skin, and nuclear morphology. Dermal collagen was capture through second harmonic generation (SHG) of imaging and was used to confirm basal stem cells where immediately adjacent to the basement membrane.

### Chromatin compaction analysis

Data analysis for the chromatin compaction plots was done in Imaris (Bitplane), FIJI (ImageJ), MATLAB, and Prism. We first surfaced the 3D volume of individual nuclei from high-resolution, intravital imaging data using default Imaris surfacing settings pixel size 0.08 µm at 0.5 µm z-slices. All voxels within the 3D volume were normalized to an 8-bit range of fluorescent intensity inherent to the individual nucleus being surfaced with the top and bottom 0.1% of voxels excluded as outliers. This allowed us to compare chromatin compaction among many different nuclei and among mouse replicates and models despite slight differences in mean fluorescent intensity. Intensity values for each voxel within the 3D volume were binned into 0–256 fluorescent intensity bins, and plotted as a percentage of total nuclear volume to account for differences in nuclear volume.

To measure the chromatin compaction within loci of active transcription, we first surfaced individual nuclei and normalized voxel intensity values as described above. We then surfaced the 3D volume of the transcriptionally active locus within each nucleus, applying the nuclear normalization to the voxel intensity values within the transcriptional locus. The intensity values for the voxels within both the nucleus and transcriptional locus were binned and plotted as described above. All custom coding scripts developed are available to reader through the Dryad data repository: https://doi.org/10.5061/dryad.5hqbzkh94.

### Topical drug treatments

To pharmacologically perturb chromatin organization, Trichostatin-A (TSA) was delivered topically to the ear skin. TSA was dissolved in a 10 mg/ml stock solution in dimethyl sulfoxide (DMSO) and then diluted 100 X in 100% petroleum jelly (Vaseline; final concentration 1 µg/ml). One hundred micrograms of the TSA/Vaseline mixture was spread evenly on the ear 48 and 24 hr before imaging. A mixture of 100% DMSO in petroleum jelly was used as a vehicle control.

### Statistics and reproducibility

Asterisks denote statistical significance (* p<0.05, ** p<0.01, *** p<0.001 and **** p<0.0001). Mean and standard deviation among mice are shown unless otherwise stated. Statistical calculations were performed using the Prism software package (GraphPad, USA).

To test statistical differences between chromatin compaction histograms, we use permutation to test the null hypothesis that the two groups have the same distribution. We define a distance between two groups of histograms. More specifically, we average histogram counts in each group and then calculate the count difference between two groups. Let $H_{k}$ be the set of all histograms in group k and ${\overset{-}{h}}_{kj}$ is the average histogram count in the interval j of group k. Then the distance is defined as

$$d\left(H_{1},H_{2}\right)=\sum_{j=1}^{J}{\left({\bar{h}}_{1j}-{\bar{h}}_{2j}\right)}^{2}\;\;\;,$$

where J is the total number of intervals. Based on this definition, we first calculate the distance between the two groups from the observed data. We then perform permutations to derive the null distribution for the distance that there is no group difference. In detail, we permute the labels of the two groups, and calculate the distance for each permuted data set. This is repeated 10,000 times to derive the histogram distance distribution empirically. Lastly, the statistical significance of the observed data is calculated by the proportion of the times that the permuted data lead to a larger distance than that observed. If the p value thus estimated is less than 0.05, we conclude that the histograms of these two groups are significantly different.

### Mouse models

K14-rtTA (Xie et al., 1999), tetO-Cdkn1b (Pruitt et al., 2013), tetO-Cre (Strain #:006234), and *R26^LSLtdTomato^ (*Madisen et al., 2010) mice were obtained from the Jackson Laboratory. K14H2B-GFP (Tumbar et al., 2004) and K14CreER (Vasioukhin et al., 1999) mice were obtained from Elaine Fuchs, *Serum Response Factor* (*Srf*) from Shangqin Guo, K10-rtTA (Muroyama and Lechler, 2017) from Terry Lechler, and MCP-GFP (Lionnet et al., 2011) from Robert Singer. *TIGRE-*K14-H2B-mCherry mice were generated by the Yale Transgenic Facility. A K14-H2B-mCherry transgene (Mesa et al., 2015) flanked by 2 X core sequence of the HS4 chicken beta globin insulator was cloned into a targeting vector (Addgene #92142) that contains homology arms of the mouse *TIGRE* genomic locus (Madisen et al., 2015). The resulting construct was then used to target into the *TIGRE* locus via CRISPR/Cas9-mediated genome editing with the gRNA, ACAGAAAACATCCCAAAGTTAGG. One correctly targeted mouse was picked for generating the stable colony.

To block the cell cycle progression of epithelial cells during G1, K14H2B-GFP mice were mated with K14rtTA; tetO-Cdkn1b mice and given doxycycline (2 mg/ml) in potable water with 2% sucrose. Doxycycline treatment was sustained until imaging was performed the next day. Siblings without the tetO-Cdkn1b allele (K14H2B-GFP; K14rtTA) were used as controls. To generate mice deficient for *Srf*, K14H2B-GFP; K14CreER mice were mated to *Srf ^fl/wt^; R26^LSLtdTomatofl/fl^* mice to generate K14H2B-GFP; K14CreER*; R26^LSLtdTomato^; Srf ^fl/wt^* and *Srf ^wt/wt^* mice in equal proportions. To visualize epidermal cells having initiated transcription of *Krt10*, we mated K14H2B-GFP; tetO-Cre mice to K10rtTA*; R26^LSLtdTomato^* mice to yield K14H2B-GFP, K10rtTA; tetO-Cre*, R26^LSLtdTomato^* mice. These mice were given doxycycline (2 mg/ml) in potable water with 2% sucrose. Doxycycline treatment was sustained until imaging was performed two days later.

*Krt10*MS2 mice were generated by the Yale Transgenic Facility. 24X MS2 repeats (Addgene #31865) were cloned into a vector containing homology arms at the first predicted high-efficiency cut site after the stop codon for the *Keratin-10* locus (26 bp after stop) (Spille et al., 2019). The resulting construct was then used to target into the keratin-10 locus via CRISPR/Cas9-mediated genome editing with the gRNA, AGTGATCAGGACGATTATTGAGG. Correctly targeted founders were identified for expansion into stable colonies. Mice were born in Mendelian ratios, and heterozygous mice for the *Krt10*MS2 allele are phenotypically normal (with normal epidermal structure) which is in agreement with literature of homozygous *Krt10* knock out mice that exhibit normal differentiation (Reichelt et al., 2001).

Mice from experimental and control groups were randomly selected for either sex for live imaging experiments. All procedures involving animal subjects were performed under the approval of the Institutional Animal Care and Use Committee (IACUC) of the Yale School of Medicine (Protocol #2021–11303).

### Tamoxifen induction

To recombine the *Srf ^fl/wt^/Srf ^wt/wt^;* K14CreER*; R26^LSLtdTomato^;* K14H2B-GFP mice, we gave a single dose of tamoxifen (20 mg/kg body weight in corn oil) by intraperitoneal injection 6 days before the final time point, immediately after imaging the day 0 timepoint.

### PHATE (Potential of Heat-diffusion for Affinity-based Transition Embedding) analysis and cell clustering

MATLAB packages were used for PHATE analysis and Louvain clustering (Moon et al., 2019; Blondel et al., 2008). PHATE was performed on a matrix composed of individual cells and 27 normalized fluorescent intensity bins. The number of diffusion steps was automatically picked and visualized using 2D PHATE embedding. The cells were clustered using the Louvain algorithm on the same matrix, where the algorithm searched for the 50 nearest neighbors based on Euclidean distance. Clusters were visualized on PHATE space.

## Data Availability

All coding scripts and source datasheets for figure quantifications are made accessible through the Dryad data repository: https://doi.org/10.5061/dryad.5hqbzkh94. Representative raw imaging data are accessible through the same link and full datasets available upon request with no restrictions (due to size) by contacting VG. Source datasheets are included in supplemental information. The following dataset was generated: MayD YunS GonzalezD ParkS ChenY LathropE CaiB XinT ZhaoH WangS GonzalezLE CockburnK GrecoV 2023Code and imaging data for: Mouse epidermal chromatin architectureDryad Digital Repository10.5061/dryad.5hqbzkh94

## Additional files

MDAR checklist
