# Supplementary material for: Live imaging reveals chromatin compaction transitions and dynamic transcriptional bursting during stem cell differentiation in vivo
Source: eLife. 2023 Mar 7;12:e83444. doi: 10.7554/eLife.83444 (PMC10027315; doi:10.7554/eLife.83444)
Supplement: Peer review history

# Editor report: Editor's evaluation

**DOI:** 10.7554/eLife.83444.sa0

**Authors:** Melike Lakadamyali

**Affiliations:** https://ror.org/00b30xv10 University of Pennsylvania United States

This is an important study that uses state-of-the-art in vivo imaging methods to advance our understanding of how chromatin architecture changes relate to the timing of transcription activation at the single-cell level in a live animal. The authors develop a quantitative framework to measure the chromatin compaction state in vivo and provide compelling evidence that chromatin compaction is heterogenous in the stem cells of the epidermis and specific to cell identity; chromatin compaction transitions over days concomitant with differentiation and transcription of an early lineage-specific gene starts prior to chromatin remodeling.

# Decision letter

**DOI:** 10.7554/eLife.83444.sa1

**Affiliations:** https://ror.org/00b30xv10 University of Pennsylvania United States

Our editorial process produces two outputs: (i) public reviews designed to be posted alongside the preprint for the benefit of readers; (ii) feedback on the manuscript for the authors, including requests for revisions, shown below. We also include an acceptance summary that explains what the editors found interesting or important about the work.

**Decision letter after peer review:**

Thank you for submitting your article "Live imaging reveals chromatin compaction transitions and dynamic transcriptional bursting during stem cell differentiation in vivo" for consideration by *eLife*. Your article has been reviewed by 2 peer reviewers, and the evaluation has been overseen by a Reviewing Editor and Didier Stainier as the Senior Editor. The reviewers have opted to remain anonymous.

The reviewers have discussed their reviews with one another, and the Reviewing Editor has drafted this to help you prepare a revised submission.

Essential revisions:

The reviewers have made suggestions about some key control experiments and clarifications, which will further strengthen the conclusions and clarity of the paper.

*Reviewer #1 (Recommendations for the authors):*

Beyond the major points made in the public review, I have no additional comments. The data is clearly presented and the manuscript is well-written.

*Reviewer #2 (Recommendations for the authors):*

1. The method to quantify chromatin compaction is a central aspect of the work. However, from the methods section, it is unclear how this analysis was performed. The authors should expand this section by describing the experimental procedure in more detail. For example, they should specify which software and software version was used at each step, as well as which plugins or packages were used. If custom-made scripts were developed, it would be important to specify them and it would be valuable to make them available to the scientific community.

2. From main Figure 3 onwards, including supplementary figures, most figure panels displaying microscopy images lack scale bars.

3. Although the authors mention which type of average and variability values were plotted (Mean +/- SD) and which test was applied in the methods section, it would be more convenient to specify this in the corresponding figure legends.

4. Are differences statistically significant for Figure 2E and Figure 3?

5. In Figure 5B the image panels are small and the quality of the signal is not very high. Bigger panels and single-color panels would help the visual interpretation of the data. As they are, transcription loci appear as big spots in H2B negative regions, they almost look like nucleoli. Also, with this system, can the authors visualize mature transcripts beside the point of nascent transcription?

6. Also, related to Keratin-10-24xMS2, only one point of active transcription is visible. Is the 24xMS2 present in only one allele or is this due to mono-allelic expression of the transcript or finally is this due to the specific focal planes where only one allele is visible? The authors should clarify this point to improve the interpretation of the results.

7. It would be valuable that the authors explain better how they can trace the same cells across different days within the complex epithelium.

# Reply: Author response

**DOI:** 10.7554/eLife.83444.sa2

Reviewer #2 (Recommendations for the authors):

1. The method to quantify chromatin compaction is a central aspect of the work. However, from the methods section, it is unclear how this analysis was performed. The authors should expand this section by describing the experimental procedure in more detail. For example, they should specify which software and software version was used at each step, as well as which plugins or packages were used. If custom-made scripts were developed, it would be important to specify them and it would be valuable to make them available to the scientific community.

We absolutely agree with all these comments. The Methods section has been expanded to reflect your recommendation and the software written in our lab are all available to the public via the Dryad repository.

2. From main Figure 3 onwards, including supplementary figures, most figure panels displaying microscopy images lack scale bars.

Thank you for this, they have all been added.

3. Although the authors mention which type of average and variability values were plotted (Mean +/- SD) and which test was applied in the methods section, it would be more convenient to specify this in the corresponding figure legends.

We have added these details to the figure legends.

4. Are differences statistically significant for Figure 2E and Figure 3?

We have added these statistics into the figures and legends.

5. In Figure 5B the image panels are small and the quality of the signal is not very high. Bigger panels and single-color panels would help the visual interpretation of the data. As they are, transcription loci appear as big spots in H2B negative regions, they almost look like nucleoli. Also, with this system, can the authors visualize mature transcripts beside the point of nascent transcription?

We have added larger panels and single-color images to Figure 5 to facilitate clearer visualization of both keratin-10 transcription loci and Keratin-10 mature transcripts. As shown in Figure 5D-E, measuring the local chromatin compaction within nuclear MS2/MCP-GFP puncta reveals that these foci are located in fluorescent intensity bins corresponding to loosely compacted, transcriptionally accessible, euchromatic regions (compare to RNA Pol II immunostaining in Figure 1D). In contrast, nucleoli are characterized by the lowest fluorescent intensity bins (Figure 1A, 5E) and are distinct from the MS2/MCP-GFP punctum (Author response image 1). We believe the K10MS2/MCP-GFP reporter system enables us to visualize mature Keratin-10 transcripts outside the nucleus, in addition to the active site of Keratin-10 transcription, consistent with implementations of the MS2/MCP tool in other systems (e.g. Dar et al., 2012; Jeziorska et al., 2022; Raj et al., 2006). Indeed, perinuclear mature Keratin-10 transcripts are detectable (Figure 5B-C), particularly in the differentiated layer where Keratin-10 is more highly expressed.

**Author response image 1.** K10MS2/MCP punctum do not colocalize with the lowest H2BGFP fluorescent regions.

6. Also, related to Keratin-10-24xMS2, only one point of active transcription is visible. Is the 24xMS2 present in only one allele or is this due to mono-allelic expression of the transcript or finally is this due to the specific focal planes where only one allele is visible? The authors should clarify this point to improve the interpretation of the results.

Thank you, we have clarified this in the text (page 17). The genotype used in Figure 5 is K14H2B-mCherry; MCP-GFP; *K10*MS2 Het. The *K10*MS2 mice were created through CRISPR insertion into the endogenous K10 locus, and so the Het mice only have one punctum of *Keratin-10* transcription.

All images were evaluated through their full z-depth as to not miss any nuclei that might have a *K10*MS2MCP punctum for the 2D insets in each figure. This will be clarified in the legends for each panel to address the point and clarify what is a max and average projection image.

7. It would be valuable that the authors explain better how they can trace the same cells across different days within the complex epithelium.

We agree that providing additional detail would be helpful. The methods section has been edited to address this, and our methods are explained in great detail in Pineda, Park, *et al.* Nature Protocols 2015. In short, we can macroscopically identify similar regions of the skin over days/weeks/months by the branched vasculature structure of the ear, and then under the microscope, we use anatomical features such as hair follicles to identify the same region of the epidermis over time where individual epidermal cells were visually compared/tracked to previous time points. (Pineda et al., 2015).

References

Kanda, T., Sullivan, K.F., and Wahl, G.M. (1998). Histone–GFP fusion protein enables sensitive analysis of chromosome dynamics in living mammalian cells. Current Biology 8, 377–385. 10.1016/S09609822(98)70156-3.

Tumbar, T., Guasch, G., Greco, V., Blanpain, C., Lowry, W.E., Rendl, M., and Fuchs, E. (2004). Defining the epithelial stem cell niche in skin. Science 303, 359–363. 10.1126/science.1092436.

Kumar, A., Maitra, A., Sumit, M., Ramaswamy, S., and Shivashankar, G.V. (2014). Actomyosin contractility rotates the cell nucleus. Sci Rep 4, 3781. 10.1038/srep03781.

Zhu, R., Liu, C., and Gundersen, G.G. (2018). Nuclear positioning in migrating fibroblasts. Seminars in Cell and Developmental Biology 82, 41–50. 10.1016/j.semcdb.2017.11.006.

Sara Gallini, Nur-Taz Rahman, Karl Annusver, David G. Gonzalez, Sangwon Yun, Catherine Matte-Martone, Tianchi Xin, Elizabeth Lathrop, Kathleen C. Suozzi, Maria Kasper, Valentina Greco. Injury suppresses Ras cell competitive advantage through enhanced wild-type cell proliferation. bioRxiv 2022.01.05.475078; doi: https://doi.org/10.1101/2022.01.05.475078

Pedro Barbacena, Marie Ouarné, Jody J Haigh, Francisca F Vasconcelos, Anna Pezzarossa, Claudio A Franco. GNrep mouse: A reporter mouse for front-rear cell polarity. Genesis 2019 Jun. DOI: 10.1002/dvg.23299

Cristiana M Pineda, Sangbum Park, Kailin R Mesa, Markus Wolfel, David G Gonzalez, Ann M Haberman, Panteleimon Rompolas, Valentina Greco. Intravital imaging of hair follicle regeneration in the mouse. Nature Protocols 2015 July. DOI: 10.1038/nprot.2015.070
